# Long-term melatonin treatment for the sleep problems and aberrant behaviors of children with neurodevelopmental disorders

**DOI:** 10.1186/s12888-020-02847-y

**Published:** 2020-09-10

**Authors:** Kotaro Yuge, Shinichiro Nagamitsu, Yuko Ishikawa, Izumi Hamada, Hiroyuki Takahashi, Hideyuki Sugioka, Osamu Yotsuya, Kazuo Mishima, Masaharu Hayashi, Yushiro Yamashita

**Affiliations:** 1 Department of Pediatrics and Child Health, Kurume University School of Medicine, 67 Asahi-machi, Kurume, Fukuoka, 830-0011 Japan; 2 Nobelpharma Co., Ltd., Tokyo, Japan; 3 CMIC Co., Ltd., Tokyo, Japan; 4 Department of Neuropsychiatry, Akita University Graduate School of Medicine, Akita, Japan; 5 Department of Nursing, College of Nursing and Nutrition, Shukutoku University, Chiba, Japan

**Keywords:** Melatonin, Neurodevelopmental disorders, Sleep onset latency, Children, Sleep problems, Aberrant behaviors, Long-term

## Abstract

**Background:**

Clinical evidence is required about the long-term efficacy and safety of melatonin treatment for sleep problems in children with neurodevelopmental disorders (NDDs) who underwent adequate sleep hygiene interventions.

**Methods:**

We conducted a 26-week, multicenter, collaborative, uncontrolled, open-label, phase III clinical trial of melatonin granules in children 6 to 15 years of age who had NDDs and sleep problems. The study consisted of the 2-week screening phase, the 26-week medication phases I and II, and the 2-week follow-up phase. Children received 1, 2, or 4 mg melatonin granules orally in the medication phases. Variables of sleep status including sleep onset latency (SOL), aberrant behaviors listed on the Aberrant Behavior Check List-Japanese version (ABC-J), and safety were examined. The primary endpoint was SOL in the medication phase I.

**Results:**

Between June 2016 and July 2018, 99 children (80 males and 19 females, 10.4 years in mean age) were enrolled at 17 medical institutions in Japan—74, 60, 22, 9, 6, and 1 of whom had autism spectrum disorder, attention-deficit/hyperactivity disorder, intellectual disabilities, motor disorders, specific learning disorder, and communication disorders, respectively, at baseline. Fifteen children received the maximal dose of 4 mg among the prespecified dose levels. SOL recorded with the electronic sleep diary shortened significantly (mean ± standard deviation [SD], − 36.7 ± 46.1 min; 95% confidence interval [CI], − 45.9 to − 27.5; *P* <  0.0001) in the medication phase I from baseline, and the SOL-shortening effect of melatonin persisted in the medication phase II and the follow-up phase. Temper upon wakening and sleepiness after awakening improved significantly (*P* <  0.0001 each) in the medication phase I from baseline and persisted in the follow-up phase. The following subscales of the ABC-J improved significantly: stereotypic behavior (*P* = 0.0322) in the medication phase I; and irritability, hyperactivity, and inappropriate speech (*P* <  0.0001) in the medication phase II. Treatment-emergent adverse events did not occur subsequent to week 16 after medication onset, and NDDs did not deteriorate in the follow-up phase.

**Conclusions:**

Long-term melatonin treatment in combination with adequate sleep hygiene interventions may afford clinical benefits to children with NDDs and potentially elevates their well-being.

**Trial registration:**

ClinicalTrils.gov, NCT02757066. Registered April 27, 2016.

## Background

Melatonin, the main hormone that is synthesized and secreted by the pineal gland of the vertebrates, has extensively been investigated in animals and humans over the last 50 years and is known for its involvement in the regulation of sleep and biorhythms [1]. In humans, melatonin is secreted soon after sunset, reaches the peak at 2 to 4 o’clock in the midnight and decreases gradually during the second half of the night [2]. Serum melatonin concentrations vary extensively between 80 to 120 pg/mL during the night and 10–20 pg/mL during daylight hours [3]. Melatonin might be the best biomarker of human circadian rhythms [4].

In children, sleep problems represent an important medical issue that physicians address frequently in the clinical settings. The proportions of children with sleep problems have ranged between 11% [5] and 40% [6]. Sleep problems of children are known to strongly impair their mental function upon wakening [7, 8] and are associated with decreases in daytime cognitive function and in school achievements [9, 10]. Furthermore, sleep problems are also known to manifest as cognitive behavioral changes (e.g., moodiness, crankiness, reduced tolerance, and difficulty maintaining attention) that resemble the signs and symptoms of attention-deficit/hyperactivity disorder (ADHD) [11]. A number of studies have reported the association of sleep problems with the signs and symptoms of neurodevelopmental disorders (NDDs) [12]. Sleep problems are most likely related to behavioral factors (e.g., inability to self-calm, anxiety, and impaired communication) [13] and to circadian sleep-wake cycle abnormalities that are attributable to abnormally low levels of melatonin [14]. Sleep patterns differed and deviated in infants with NDDs compared to their unaffected counterparts, with developmental inconsistencies in sleep-wake state organization during the infancy period [15, 16]; however, the association was not recognized by Scher et al. [17]. Furthermore, sleep problems of children not only cause the mental and physical burdens for their caregivers and family members [18], but also influence their school attendance as part of communal living [19]. Wasdell et al. conducted a randomized, placebo-controlled, double-blind, crossover trial of controlled-release melatonin in children with NDDs and described seizures, infection, gastrointestinal illness, and agitation as the most common adverse events (AEs) [20]. Melatonin causes generally minor, short-lived, and easily manageable AEs, most of which are related to fatigue, mood, or psychomotor and neurocognitive performance [21].

The biosynthesis and secretion of melatonin are controlled by the light-dark cycle [22, 23], and melatonin secretion and sleep time in humans respond to changes in day length [24]. An abundant body of research evidence demonstrates that exogenous melatonin is effective for the treatment of sleep problems [2, 25–32]. Melatonin induces sleep when the homeostatic mechanism of sleep is insufficient and suppresses the arousal desire generated by the pacemaker of the circadian rhythms, thus shifting them in such a manner that the sleep-prone phase of the circadian rhythms occurs at a newly desired time. Therefore, exogenous melatonin can act as a hypnotic, chronohypnotic, or chronobiotic drug.

Melatonin is not approved as a pharmaceutical in Japan. In Europe, however, melatonin—approved in 2007 for the treatment of “primary insomnia of individuals 55 years or older”—was marketed by Neurim Pharmaceuticals, Israel, under the trademark of Circadin. In the United States, a number of melatonin products are commercially available as supplements but not as approved drugs. In Japan, there are great clinical needs for not-yet-approved melatonin as evidenced by its tentative use at hospitals and clinics, as well as by private importing. Melatonin prolonged-release tablets were approved by the European Medicines Agency in September 2018. Slenyto is indicated for the treatment of insomnia in children and adolescents aged 2–18 with autism spectrum disorder and/or Smith-Magenis syndrome, where sleep hygiene measures have been insufficient.

The objective of the present clinical trial was to examine the long-term efficacy and safety of melatonin granules that were administered orally to children with NDDs.

## Methods

### Subjects

A child or adolescent was considered eligible when meeting the following inclusion criteria at the time of consent/assent acquisition or at the onset of the screening phase: 1) 6 to 15 years of age; 2) meeting the diagnostic criteria of NDDs defined in Diagnostic and Statistical Manual of Mental Disorders, Fifth Edition (DSM-5) [33]—intellectual disabilities, communication disorders, autism spectrum disorder (ASD), ADHD, specific learning disorder, motor disorders, and other NDDs, with heed to the aftermentioned “exclusion criteria 1)” regarding intellectual disabilities; 3) the mean of sleep onset latency (SOL: time from bedtime to time of falling asleep, lasting for 30 or more minutes) in daily life persisted for 3 or more months; 4) caregivers and their child (to the extent of their comprehension) can comprehend what to do through sleep hygiene interventions and medication teaching; 5) caregivers can be cooperative for monitoring their child’s sleep status and entering required information in the electronic sleep diary; 6) caregivers and their child can visit the hospital as scheduled; 7) being an outpatient; and 8) prior to participation in the present clinical trial, the surrogate consenter can give written consent or the child can give consent or assent. Furthermore, a child or adolescent was considered eligible when meeting the following inclusion criteria at the completion of the screening phase: 9) SOL lasting for 30 or more minutes persisted for 3 or more days among the last 7 days during the screening phase; 10) adherence to medication time and bedtime that were prespecified at the onset of the screening phase, with allowances of ±30 min and ± 60 min for medication time and bedtime, respectively—2 or less “out-of-allowance days” among the last 7 days during the screening phase (with the exception of the day when the child fell asleep prior to medication); 11) appropriate entry of required information in the electronic sleep diary—2 or less “missing-of-entry days” among the last 7 days during the screening phase; and 12) 2 or less “nonmedication” days of the last 7 days during the screening phase (with the exception of the day when the child fell asleep prior to medication). The key exclusion criteria were as follows: 1) intellectual disabilities that were categorized to “severest” or greater in DSM-5 severity regarding one or more conceptual, social, or practical domains; 2) history of melatonin use (including supplements); 3) ramelteon use within 4 weeks prior to the onset of the screening phase; 4) a history of hypersensitivity or allergy to ramelteon; 5) the use of three or more drugs to treat epilepsy; and 6) comorbidity—schizophrenia or bipolar disorder. The present clinical trial was registered at ClinicalTrials.gov (NCT02757066).

### Study design

A multicenter, collaborative, uncontrolled, open-label, phase III clinical trial consisted of the 2-week screening phase, the 26-week medication phases (phase Ι: 10 weeks; phase II: 16 weeks), and the 2-week follow-up phase. In the medication phases, 1, 2, or 4 mg melatonin granules were administered orally once daily before bedtime, starting at 1 mg per day; the investigator increased or reduced the dose at his/her discretion based on the clinical condition of the child. In the screening phase, placebo was orally administered in an unmasked manner and children were checked for history of present illness, anamnesis, previous history of treatment, comorbidities, concurrent drugs, concurrent therapies, inclusion criteria, exclusion criteria, sleep hygiene intervention, electronic sleep diary, aberrant behavior checklist-Japanese version (ABC-J), prescription of the investigational drugs, status of medication, height, body weight, and vital signs. Nobelpharma Co., Ltd.—the study sponsor—delivered to each of 17 medical institutions 210 packs per one child, each of which contained 1 mg of 0.2% granules consisting of synthesized melatonin and of mannitol as the excipient and which were inserted in boxes.

### Primary and secondary endpoints

The primary endpoint was SOL recorded with the electronic sleep diary in the medication phase I—defined as the difference in the median SOL during 7 days before the completion of the medication phase I or medication discontinuation from baseline that was defined as the median SOL during 7 days just before the onset of the screening phase. The secondary endpoints were as follows: 1) time from the onset of medication to medication suspension; 2) SOL at the time of medication suspension; 3) time from medication suspension to medication resumption; 4) items recorded with the electronic sleep diary—number of awakenings after sleep onset, time of falling asleep, wakening time, awakening time, refusal to going to bed at prespecified bedtime, temper upon wakening, and sleepiness intensity after awakening; and 5) ABC-J.

### Sleep hygiene interventions

Investigators used the sponsor-prepared leaflet to conduct adequate sleep hygiene interventions when guiding caregivers and their child to gain favorable sleep habits and improving living environments. Nobelpharma prepared the illustrated leaflet easy to read for children in order to explain the following instructions: 1) Receive sunlight in the morning by opening the curtains so that you may wake up smoothly in both body and mind; 2) Take breakfast that is the source of power for increasing body temperature and energetic activities for the day; 3) Move your body during the day so that you may get tired pleasantly and fall asleep quickly through the actions of melatonin during the night; and 4) Go to bed early and turn off light in bed (be careful not to watch television or play games too much). Caregivers were provided with the electronic sleep diary to record bedtime of their child during the study period. In consideration of the living environments of the child and of the extent of his/her adhesion to the interventions during the study period, target bedtime was accelerated by ≤2 h earlier than the “average bedtime in daily living” before participation. Medication time was set to 45 min before bedtime. Caregivers and their child were instructed to adhere to prespecified bedtime and medication time during the screening phase and the medication phase I. Between week 10 of the medication phase I and week 22 of the medication phase II, bedtime could be accelerated (1 h maximum per acceleration) in consideration of the sleep status of the child and of their caregivers’ desire. The modification of bedtime in the medication phase II was not restricted to ≤2 h earlier than the “average bedtime” of the child.

### Comedications

Except topical agents, the administration of the following comedications was prohibited as of 4 weeks prior to the onset of the screening phase: melatonin receptor agonists (e.g., melatonin and ramelteon), hypnotics and sedatives (e.g., triclofos), hypnotics (e.g., triazolam), antiparkinsonians (e.g., levodopa), psychoanaleptics (e.g., pemoline), first-generation antihistamines (e.g., diphenhydramine hydroxide), caffeine-containing agents (e.g., caffeine hydroxide), and fluvoxamine maleate. The administration of the following comedications was allowed when administered as of 4 weeks prior to the onset of the screening phase and when not modifying their dosage and administration during the study period: drugs to treat ADHD (e.g., methylphenidate), vitamin B_12_, antipsychotics (e.g., risperidone), anxiolytics (e.g., diazepam), antidepressants (e.g., amoxapine), antimaniac drugs (e.g., carbamazepine), and traditional Japanese Kampo medicine (e.g., yoku-kan-san).

### Electronic sleep diary and questionnaires

From the night of the onset of the screening phase to the night of the completion of the follow-up phase, caregivers used the electronic sleep diary to record medication time, bedtime, time of falling asleep, wakening time, awakening time, number of wakenings after sleep onset, refusal to going to bed at prespecified bedtime, temper upon wakening, and sleepiness intensity after awakening.

Physicians and clinical research associates used the ABC-J—a 58-item questionnaire of 4-level scale consisting of the following 5 subscales: “irritability,” “lethargy,” “stereotypic behavior,” “hyperactivity,” and “inappropriate speech”—to investigate the aberrant behaviors of the child. The ABC was developed by Aman et al. [34, 35] to assess the effects of pharmacotherapy on the aberrant behaviors of individuals with intellectual disabilities. The ABC-J was developed by Ono et al. [36], whose usefulness to assess the effects of psychotropics on individuals with intellectual disabilities was demonstrated based on reliability and adequacy.

### Safety

The safety of long-term melatonin treatment was assessed based on the incidences of AEs and treatment-emergent adverse events (TEAEs), laboratory tests including hematology (e.g., white blood cell counts, red blood cell counts, and hemoglobin concentration), blood chemistry (e.g., albumin, total protein, and alkaline phosphatase), urinalysis (e.g., specific gravity, pH, and glucose), and 12-lead resting electrocardiography, vital signs—blood pressures, pulse rate, and body temperature—, height, body weight, rebound phenomenon, and withdrawal symptoms. The preferred terms listed on the Japanese version of the Medical Dictionary for Regulatory Activities version 19.0 [37] were used to express AEs and TEAEs.

### Statistical analysis

Continuous variables are expressed as mean ± SD, and categorical variables as medians with the interquartile range. The primary and subgroup analyses of the efficacy of melatonin treatment in the full analysis set were conducted according to Wilcoxon’s signed rank sum test. A 5% two-sided *P* value of < 0.05 was considered statistically significant. All statistical analyses were conducted using the SAS software (SAS Institute, Cary, NC, USA) version 9.3.

## Results

### Subject disposition

**Fig. 1:**
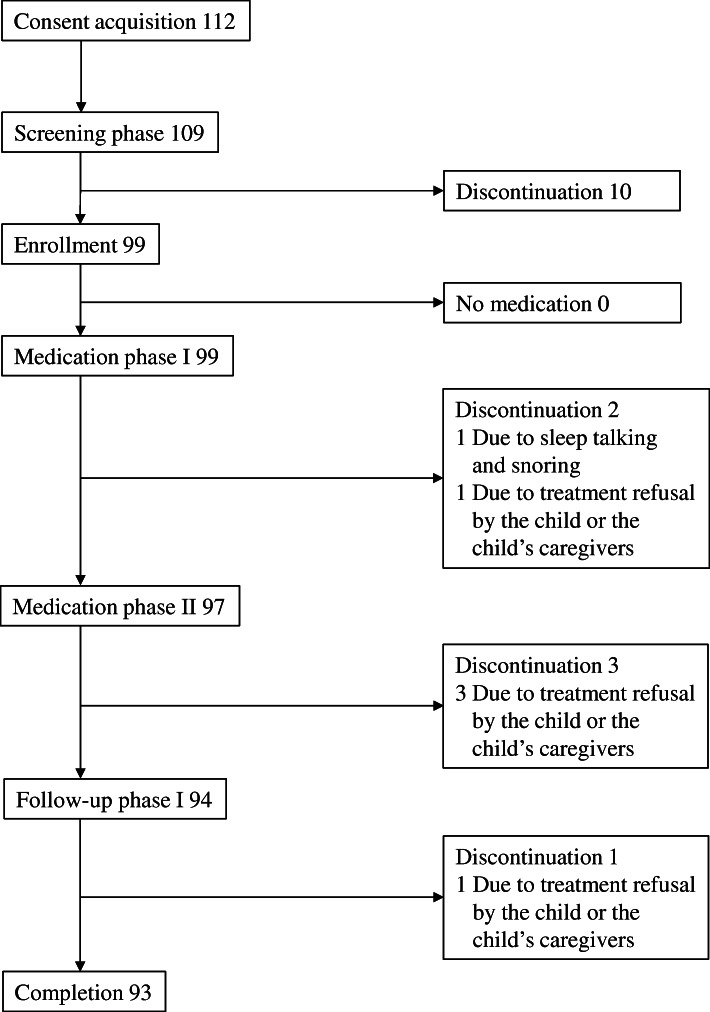
Subject disposition

Between June 2016 and July 2018, 99 children (80 males and 19 females, mean age: 10.4 years) were enrolled at 17 medical institutions in Japan. Subject disposition is shown in Fig. 1. Written informed consent/assent was obtained from 112 caregivers/17 surrogate consenters or children. Among 109 screened children, 99 entered the medication phase I and orally received melatonin granules. In the screening phase, 10 children were excluded due to ineligibility (i.e., unmet inclusion criteria and falling under exclusion criteria). Two children were excluded during the medication phase I: one due to the child’s or caregivers’ proposal for discontinuation because of sleep talking and snoring and another due to treatment refusal by the child or the child’s caregivers. Consequently, 97 children made the transition to the medication phase II. Three children were excluded during the medication phase II due to treatment refusal by them or their caregivers. Consequently, 94 children made the transition to the follow-up phase. One child was excluded during the follow-up phase due to treatment refusal by the child or the child’s caregivers. Consequently, 93 of 99 children (93.9%) completed the present clinical trial.

### Background of children and adolescents

Table 1 summarizes the demographic and clinical characteristics of children at baseline. Males were predominant (80.8%), with the mean age of 10.4 years and the prevailing proportion of children 6 to 11 years of age (63.6%). The mean body weight was 37.1 kg, with the predominating proportion of children of ≥30 kg in body weight (62.6%). Children and adolescents who had concurrent diseases accounted for 82.8% (82/99), including allergic rhinitis (26.3%: 26/99), seasonal allergy (18.2%: 18/99), asthma (10.1%: 10/99), allergic conjunctivitis and food allergy (8.1% each: 8/99 each), constipation (7.1%: 7/99), headache and strabismus (6.1% each: 6/99 each), enuresis and epilepsy (5.1% each: 5/99), and other comorbidities of < 5% in incidence (e.g., atopic dermatitis, eczema, and obesity).

**Table 1:** Demographic and clinical characteristics of children and adolescents with neurodevelopmental disorders at baseline

Characteristics	Categories	N (99)
		n (%)
Sex	Male	80 (80.8)
	Female	19 (19.2)
Age, yrs	Mean ± SD	10.4 ± 2.5
	6–11	63 (63.6)
	12–15	36 (36.4)
Body weight, kg	Mean ± SD	37.1 ± 15.1
	< 30	37 (37.4)
	≥ 30	62 (62.6)
	< 20	6 (6.1)
	20–29	31 (31.3)
	30–39	26 (26.3)
	40–49	18 (18.2)
	50–59	10 (10.1)
	≥ 60	8 (8.1)
Height, cm	Mean ± SD	140.3 ± 17.1
	< 100	0 (0.0)
	100–114	8 (8.1)
	115–129	25 (25.3)
	130–144	26 (26.3)
	145–159	26 (26.3)
	160–174	14 (14.1)
	≥ 175	0 (0.0)
Complications	Absent	17 (17.2)
	Present	82 (82.8)
History of ramelteon treatment	Absent	75 (75.8)
	Present	24 (24.2)
Intellectual disabilities	Absent	77 (77.8)
	Present	22 (22.2)
Communication disorders	Absent	98 (99.0)
	Present	1 (1.0)
Autism spectrum disorder	Absent	25 (25.3)
	Present	74 (74.7)
Attention-deficit/hyperactivity disorder	Absent	39 (39.4)
	Present	60 (60.6)
Specific learning disorder	Absent	93 (93.9)
	Present	6 (6.1)
Motor disorders	Absent	90 (90.9)
	Present	9 (9.1)
Other neurodevelopmental disorders	Absent	99 (100.0)
	Present	0 (0.0)

Values are expressed as mean ± SD

The proportion of children who had never been treated with ramelteon was as high as 75.8%. Among NDDs, ASD and ADHD accounted for 74.7 and 60.6%, respectively; the proportion of children who had both ASD and ADHD was 40.4%. The means of age, body weight, and height tended to increase slightly along with increments in maximal doses—1, 2, and 4 mg: for age, 9.9, 10.8, and 11.1 years, respectively; for body weight, 33.9, 39.2, and 41.8 kg, respectively; and for height, 135.8, 143.3, and 146.3 cm, respectively. Namely, little changes in the means of age, body weight, and height were found as compared with dose increments.

### Efficacy

#### Primary endpoint

**Fig. 2:**
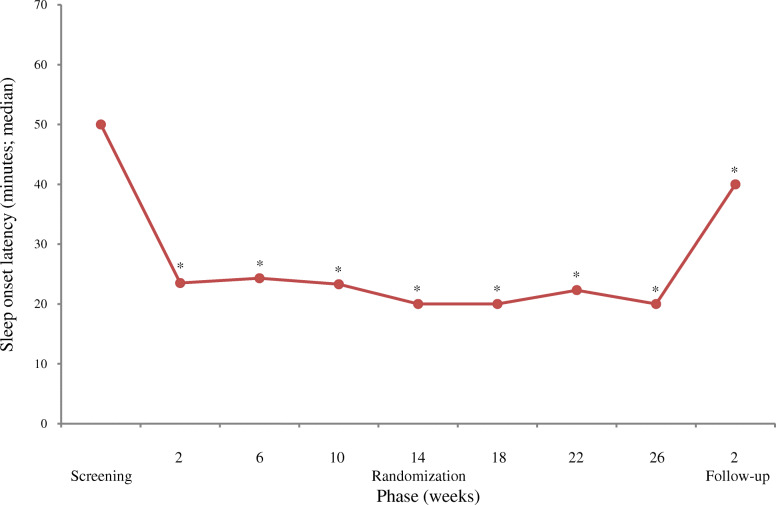
Changes in sleep onset latency recorded with the electronic sleep diary. *: *P* <  0.0001 (vs. screening) according to Wilcoxon’s signed rank sum test

The primary analysis of the primary endpoint, SOL in the medication phase I, revealed a significant shortening (median, − 30.0 min; Q1–Q3, − 46.0 to − 15.0; Wilcoxon’s signed rank sum test; *P* <  0.0001). The secondary analysis of the primary endpoint indicated a quick and significant shortening (*P* <  0.0001) at week 2 of the medication phase I from baseline, with a relatively narrow variation range of − 27.5 to − 31.5 min between week 2 (medication phase I) and week 26 (medication phase II). Furthermore, a significant shortening (*P* = 0.0012) persisted until week 2 of the follow-up phase (Fig. 2). The median changes in SOL in children who received the final doses of 1 mg (*n* = 49), 2 mg (*n* = 31), and 4 mg (*n* = 12) per day were − 32.0 min, − 41.0 min, and − 24.0 min, respectively. The median changes in SOL according to the 10-kg body weight categories were examined. Consequently, the minimum of the median changes in SOL (− 22.0 min) was found in the 30–39 kg category, and the maximum of the median changes in SOL (− 45.0 min) was noted in the 50–59 kg category. Any given tendency was not detected in the median changes between SOL and body weight.

The primary endpoint was analyzed with respect to the following subgroups: sex, male/female; age at the time of consent acquisition, 6–11/12–15 years; body weight at the onset of the screening phase, < 30/≥ 30 kg; history of ramelteon treatment, absent/present; maximal doses, 1/2/4 mg; intellectual disabilities, absent/present; communication disorders, absent/present; ASD, absent/present; ADHD, absent/present; specific learning disorder, absent/present; motor disorders, absent/present; and other NDDs, absent/present. Consequently, the scores of the subgroups—ASD, ADHD, intellectual disabilities, motor disorders, and specific learning disorder—in the medication phase I reduced significantly (*P* <  0.0001) from baseline (Table 2). All other subgroups showed no great changes in any categories examined.

**Table 2:** Subgroup analyses of changes in sleep onset latency recorded with the electronic sleep diary

Subgroups	Phases	Changes^a^			Wilcoxon’s signed rank sum test
		n	Mean ± SD	95% CI	*P* value
Autism spectrum disorder					
Absent	Screening phase	–	–	–	–
	Medication phase I	25	−36.2 ± 40.5	−53.0 to −19.5	< 0.0001
Present	Screening phase	–	–	–	–
	Medication phase I	74	−36.8 ± 48.1	−48.0 to −25.7	< 0.0001
ADHD					
Absent	Screening phase	–	–	–	–
	Medication phase I	39	−42.7 ± 62.5	−62.9 to −22.4	< 0.0001
Present	Screening phase	–	–	–	–
	Medication phase I	60	−32.8 ± 31.2	−40.9 to −24.7	< 0.0001
Intellectual disabilities					
Absent	Screening phase	–	–	–	–
	Medication phase I	77	−35.8 ± 44.6	−46.0 to −25.7	< 0.0001
Present	Screening phase	–	–	–	–
	Medication phase I	22	−39.7 ± 52.1	−62.8 to −16.6	< 0.0001
Motor disorders					
Absent	Screening phase	–	–	–	–
	Medication phase I	90	−37.4 ± 47.9	−47.4 to −27.3	< 0.0001
Present	Screening phase	–			
	Medication phase I	9	−29.8 ± 19.7	−45.0 to −14.6	0.0039
Specific learning disorder					
Absent	Screening phase	–	–	–	–
	Medication phase I	93	−36.0 ± 47.1	−45.7 to −26.3	< 0.0001
Present	Screening phase	–	–	–	–
	Medication phase I	6	−46.8 ± 25.3	−73.4 to −20.3	0.0313

^a^: Change = (median during the last 7 days of the medication phase I) - (median during the last 7 days of the screening phase)

*SD* Standard deviation, *CI* Confidence interval, *ADHD* Attention-deficit/hyperactivity disorder

#### Secondary endpoints

The median changes in time of falling asleep ranged between − 31 to − 32 min and − 38 to − 44 min at weeks 2 (medication phase I) and 26 (medication phase II), respectively; these changes were statistically significant (*P* <  0.0001) against baseline. The medians of the recorded times of falling asleep in the screening phase and the medication phases I (week 10) and II (week 26) were 22:55, 22:15, and 22:05, respectively. Thus, time of falling asleep tended to be accelerated during the medication phases. These changes were statistically significant against baseline in the medication phases I and II (*P* <  0.0001), as well as in the follow-up phase (*P* = 0.0007) (data not shown).

**Fig. 3:**
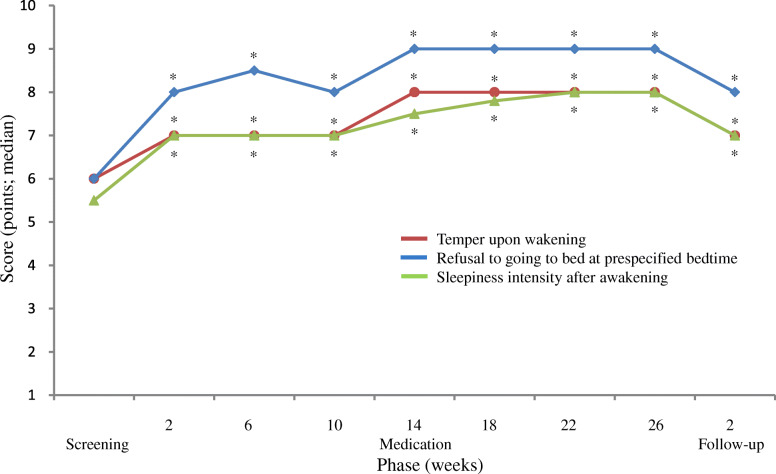
Changes in the scores of temper upon wakening, refusal to going to bed at prespecified bedtime, and sleepiness intensity after awaking calculated based on the electronic sleep diary. *: *P* <  0.0001 (vs. screening) according to Wilcoxon’s signed rank sum test

The scores of refusal to going to bed at prespecified bedtime, of temper upon wakening, and of sleepiness intensity after awakening in the screening phase, the medication phases I (week 10) and II (week 26), and the follow-up phase improved significantly (*P* <  0.0001 to *P* = 0.0014) from baseline (Fig. 3), with the medians of 6.0, 8.0, 9.0, and 8.0 points, respectively, for the former variable; those of 6.0, 7.0, 8.0, and 7.0 points, respectively, for the middle variable; and those of 5.5, 7.0, 8.0, and 7.0 points for the latter variable. Improvements as good as those found in the medication phase I (week 10) were maintained also in the follow-up phase.

Time to “the day when the investigator considered medication suspension allowable” as determined based on the improved sleep status was 112.7 ± 37.4 days in three children whose dose on day before medication suspension was 1 mg. Time to medication suspension due to an AE was 127.0 days in one child whose dose on day before medication suspension was 1 mg. SOL in three children about whom the investigator considered medication suspension allowable, whose dose on day before medication suspension was 1 mg, was 19.0 ± 9.6 min. Furthermore, SOL in one child, whose dose on day before medication suspension was 1 mg, was 10.0 min. Time between the day when the investigator considered medical suspension allowable and the day of medication resumption was 38.0 days in one child, while time between medication suspension due to an AE and medication resumption was 29.0 days in another child. Furthermore, two children showing medication suspension due to an AE made the transition to the follow-up phase without resuming medication. Changes in the number of awakenings after sleep onset reduced significantly (*P* = 0.0413 and *P* = 0.0358) at weeks 18 and 26 of the medication phase II. However, the median numbers of awakenings after sleep onset in the screening phase was low, being 0.0 episode before the transition to the medication phase I. The median changes in wakening time ranged between 0 to − 8 min, showing significant reduction at week 2 of the medication phase I (*P* = 0.0054) and at week 26 of the medication phase II (*P* = 0.0200) from baseline. However, wakening time did not show any obvious change in other phases. The median changes in awakening time ranged between − 5 to − 15 min at week 3 of the medication phase I and at week 26 of the medication phase II, showing significant reductions (*P* = 0.0147 to *P* <  0.0001) in any phases from baseline. The adequate sleep hygiene interventions accelerated sleep time as follows: 23:07, 22:26, and 22:17 in the screening phase, the medication phase I, and the medication phase II, respectively.

**Fig. 4:**
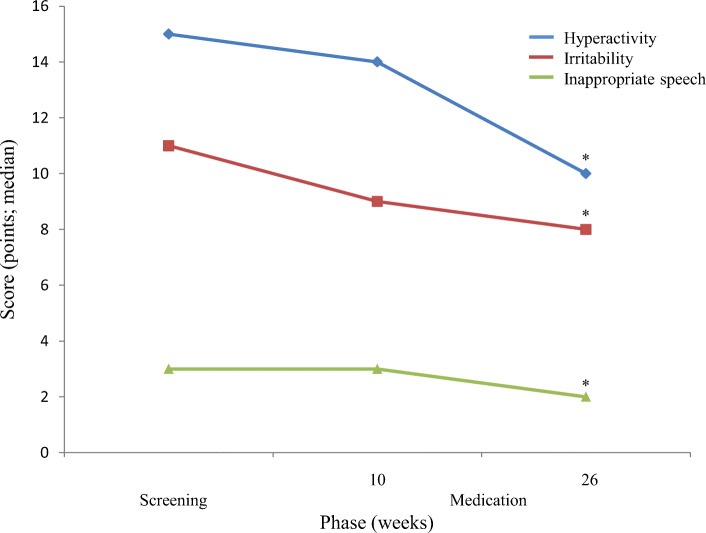
Changes in the scores of hyperactivity, irritability, and inappropriate speech calculated based on the ABC-J. *: *P* <  0.0001 (vs. screening) according to Wilcoxon’s signed rank sum test. ABC-J, the aberrant behavior check list Japanese version

Changes in aberrant behaviors of children assessed with the ABC-J are summarized in Table 3 and Fig. 4. Subscale III (stereotypic behavior; *P* = 0.0322) improved significantly at week 10 of the medication phase I from baseline, and subscales I (irritability; *P* = 0.0094), IV (hyperactivity; *P* = 0.0025), and V (inappropriate speech; *P* = 0.0125) improved significantly at week 26 of the medication phase II from baseline. Subscale II (lethargy) did not change significantly in any phases.

**Table 3:** Changes in aberrant behaviors of children assessed with the ABC-J

Subscales	Visits^a^	Changes^b^				Wilcoxon’s signed rank sum test^c^
		n	Q1	Median	Q3	*P* value
I (irritability)	2	–	–	–	–	–
	5	99	−4	0.0	2	0.1197
	9	97	−5	−1.0	2	0.0094
II (lethargy)	2	–	–	–	–	–
	5	99	−3	0.0	3	0.5533
	9	97	−4	−1.0	3	0.1267
III (stereotypic behavior)	2	–	–	–	–	–
	5	99	−2	0.0	0	0.0322
	9	97	−2	0.0	1	0.1035
IV (hyperactivity)	2	–	–	–	–	–
	5	99	−5	0.0	4	0.5504
	9	97	−6	−1.0	2	0.0025
V (inappropriate speech)	2	–	–	–	–	–
	5	99	−1	0.0	1	0.3078
	9	97	−2	0.0	1	0.0125

^a^: visit 2 = end of the screening phase; visit 5 = week 10 of the medication phase II; visit 9 = week 26 of the medication phase II

^b^: Change = (measured value at each visit) - (measured value at visit 2)

^c^: Comparison against visit 2

*ABC-J* Aberrant Behavior Check List-Japanese version

### Safety

The safety of melatonin was assessed in 99 children who constituted the safety population, and AEs in the medication phases I (*N* = 99) and II (*N* = 97) are summarized in Table 4. Among AEs that occurred in 81 children (81.8%), 14 (14.1%) were considered TEAEs. AEs in the medication phases I and II were mild, moderate, and severe in 75 children (75.8%), five children (5.1%), and one child (1.0%), respectively. All of TEAEs were mild; notably, TEAEs did not occur subsequent to week 16 after medication onset. NDDs did not deteriorate in the follow-up phase. A severe AE in one child was facial bone fracture, AEs leading to medication discontinuation in one child were blood potassium increased and headache, as well as AEs leading to medication discontinuation in one child were sleep taking and snoring in one child and those in another child were facial bone fracture and contusion; all these AEs were assessed to be “unrelated” to the medication based on the final clinical outcomes. Moderate AEs occurred in one child each, i.e., gastroenteritis, influenza, ADHD, irritability, and wound, while a severe AE—fracture of the facial bone—occurred in one child. All of these AEs were assessed to be “unrelated” to the medication. AEs (incidence: ≥ 2%) occurred in 97 children (98.0%) in the medication phase II; bronchitis was most predominant (49.5%), followed by nasopharyngitis (23.7%) and influenza (9.3%). No large differences in the incidences of AEs and TEAEs were noted with respect to the subgroups—sex, age, body weight, history of ramelteon treatment, intellectual disabilities, ASD, and ADHD.

**Table 4:** Adverse events (incidence: ≥ 2%)

	Medication phase I		Medication phase II	
	*N* = 99		*N* = 97	
	Episodes, n	Cases, n (%)	Episodes, n	Cases, n (%)
	133	59 (59.6)	166	66 (68.0)
Infections and infestations	56	39 (39.4)	72	48 (49.5)
Bronchitis	3	3 (3.0)	1	1 (1.0)
Gastroenteritis	5	5 (5.1)	6	5 (5.2)
Influenza	4	4 (4.0)	9	9 (9.3)
Nasopharyngitis	22	17 (17.2)	34	23 (23.7)
Otitis media	1	1 (1.0)	2	2 (2.1)
Pharyngitis	5	5 (5.1)	9	8 (8.2)
Upper respiratory tract infection	5	5 (5.1)	3	3 (3.1)
Neoplasms benign, malignant and unspecified	0	0 (0.0)	2	2 (2.1)
Skin papilloma	0	0 (0.0)	2	2 (2.1)
Immune system disorders	2	2 (2.0)	2	2 (2.1)
Hypersensitivity	2	2 (2.0)	0	0 (0.0)
Seasonal allergy	0	0 (0.0)	2	2 (2.1)
Metabolism and nutrition disorders	4	4 (4.0)	0	0 (0.0)
Decreased appetite	2	2 (2.0)	0	0 (0.0)
Psychiatric disorders	4	4 (4.0)	7	7 (7.2)
Attention deficit/hyperactivity disorder	0	0 (0.0)	2	2 (2.1)
Irritability	0	0 (0.0)	3	3 (3.1)
Nervous system disorders	6	5 (5.1)	10	9 (9.3)
Headache	4	3 (3.0)	4	3 (3.1)
Somnolence	2	2 (2.0)	4	4 (4.1)
Respiratory, thoracic, and mediastinal disorders	10	8 (8.1)	10	6 (6.2)
Epistaxis	3	1 (1.0)	5	2 (2.1)
Rhinitis allergic	2	2 (2.0)	2	2 (2.1)
Upper respiratory tract inflammation	3	3 (3.0)	1	1 (1.0)
Gastrointestinal disorders	22	15 (15.2)	25	12 (12.4)
Abdominal pain	6	4 (4.0)	0	0 (0.0)
Constipation	3	3 (3.0)	3	3 (3.1)
Enterocolitis	2	2 (2.0)	0	0 (0.0)
Nausea	2	2 (2.0)	0	0 (0.0)
Stomatitis	3	3 (3.0)	4	3 (3.1)
Vomiting	3	3 (3.0)	9	3 (3.1)
Skin and subcutaneous tissue disorders	10	7 (7.1)	12	8 (8.2)
Dermatitis	0	0 (0.0)	2	2 (2.1)
Eczema	1	1 (1.0)	2	2 (2.1)
Rash	2	2 (2.0)	0	0 (0.0)
Urticaria	2	1 (1.0)	3	2 (2.1)
Renal and urinary disorders	2	2 (2.0)	0	0 (0.0)
Proteinuria	2	2 (2.0)	0	0 (0.0)
General disorders and administration site conditions	3	3 (3.0)	3	3 (3.1)
Pyrexia	2	2 (2.0)	1	1 (1.0)
Investigations	7	5 (5.1)	2	2 (2.1)
Weight increased	0	0 (0.0)	2	2 (2.1)
Urobilinogen urine	2	2 (2.0)	0	0 (0.0)
Injury, poisoning and procedural complications	5	5 (5.1)	14	12 (12.4)
Arthropod sting	0	0 (0.0)	4	4(4.1)
Contusion	2	2 (2.0)	5	5(5.2)
Wound	1	1 (1.0)	3	3(3.1)

No death occurred during the medication and follow-up phases. AEs occurred in 22 children in the follow-up phase, with no large differences in contents as compared with those found in the medication phases I and II. These AEs were assessed to be “unrelated” to the medication. Seven episodes of AEs leading to the dose reduction of melatonin occurred in seven children: four episodes of somnolence; and one episode each of headache, nausea, and irritability. All of these children completed the medication phases I and II and made the transition to the follow-up phase. However, medication was discontinued for one subject who had somnolence due to a reason other than AEs in the second half of the follow-up phase. Laboratory tests revealed 13 episodes of slightly abnormal laboratory values in 10 children as follows: three cases of weight gain; two cases each of increases in hepatic function variables and urinary urobilinogen; one case each of transaminase increased, blood potassium increased, blood triglyceride increased, eosinophil count increased, urinary pH increased, and urinary specific gravity increased. All these abnormal changes resolved or recovered without requiring treatment. Any changes of special note were not found with respect to vital signs, physical findings, and other monitoring items that are related to the safety of melatonin treatment. Neither withdrawal symptoms nor rebound phenomenon were detected in the follow-up phase. The incidences of AEs and TEAEs did not change extensively after the onset of melatonin treatment.

## Discussion

This 26-week, open-label clinical study showed that immediate-release melatonin was effective in shortening SOL, the primary efficacy endpoint of the study, and was safe for children with NDDs. The efficacy of melatonin in studied children was established as early as week 2 of medication, lasted until week 26 of medication, and quickly reduced while maintaining a significant difference from the baseline value. On the other hand, Maras et al. [38] conducted a long-term open-label study of pediatric-appropriate, prolonged-release melatonin (PedPRM) in children with ASD and Smith-Magenis syndrome (SMS); they showed that the efficacy of PedPRM appeared at week 3 of medication and lasted until week 52 of follow-up. These findings drive us to consider that that the appearance and duration profile of melatonin’s efficacy are minimally influenced by dosage formulations (immediate- and prolonged-release) or target NDDs. Subsequent to the introduction of DSM-5 in 2013 [33], this is the first long-term (26-week) clinical study using immediate-release melatonin that showed the SOL-shortening effect in 5 clinicopathological entities of NDDs (i.e., ASD, ADHD, intellectual disabilities, motor disorders, and specific learning disorder), as compared with 2 clinical studies that investigated 2 equivalents of NDDs: ASD and SMS [38, 39]. Of special note were the facts that as high as 22.2% of studied children had DSM-5-defined intellectual disorders that are generally considered difficult to assess clinically and that no TEAEs occurred subsequent to week 16 after medication onset. Furthermore, melatonin improved children’s well-being as assessed with the qualitative variables (i.e., temper upon wakening, refusal to going to bed at prespecified bedtime, and sleepiness intensity after awakening), as well as the cardinal subscales of aberrant behaviors—irritability, hyperactivity, and inappropriate speech. We speculate that shortened SOL (− 30 min in the medication phase I from the screening phase), accelerated sleep time (50 min in the medication phase II), and persisting efficacy for temper upon wakening in the follow-up phase translated into the improved well-being of children, which exerted a beneficial impact on their aberrant behaviors (especially, irritability and hyperactivity) and which eventually led to reductions in family’s distress and to clinically relevant improvements in caregiver’s sleep and quality of life. Our data corroborate the clinical study of Maras et al. [38] in that improving the sleep of children with sleep problems is important for the alleviation of their family’s distress.

In the present study, we decided not to assess another major sleep parameter frequently used in the clinical studies of children with NDDs—total sleep time (TST)—based on the following arguments: 1) the 26-week precise measurement of TST requires the precisely measured wakening time after sleep onset, which implies an unacceptably increased burden for caregivers; 2) the precision of TST measurements is greatly influenced by parent-child relationships, age, intellectual level, and habitation environments for sleep, which results in a considerable difference in capturing the child’s wakening time; and 3) the clinical benefits of melatonin treatment as pharmacotherapy might be greater for both children and caregivers when improvements are obtained in SOL, qualitative variables of well-being (e.g., temper upon wakening, refusal to going to bed at bedtime, and sleepiness intensity after awakening), and aberrant behaviors (e.g., irritability and hyperactivity) rather than TST. In addition to these arguments, current treatments for aberrant behaviors (e.g., cognitive behavioral therapy, pharmacotherapy, as well as home and school support) [40–42] have provided limited clinical outcomes, suggesting a need for adopting a perspective that is different from the current therapeutic approaches. In this regard, melatonin therapy in combination with adequate sleep hygiene interventions may build a momentum for a radical shift in the current therapeutic modalities for NDDs.

Melatonin caused neither serious AEs, nor unexpected AEs. Moreover, the majority of AEs were mild in intensity and low in incidence, and the most predominant AEs were infections and infestations including bronchitis and nasopharyngitis that are frequently seen in children of ages examined. The good tolerability of children to melatonin treatment in the present study is consistent with an abundant body of clinical evidence obtained in a systemic review that analyzed 13 randomized clinical trials (*N* = 682) and in a meta-analysis that analyzed 9 studies (*N* = 541) [43]. In the present study, concretely, 94 among 99 children completed medication, only one child discontinued medication due to a TEAE, and none of them discontinued medication due to the lack of efficacy. We speculate that the dosing scheme using 1, 2, or 4 mg of melatonin granules, flexibly adjusted on an individual child basis, may have contributed to no occurrence of TEAEs subsequent to week 16 after medication onset.

In recent years, Gringras et al. [39] conducted a 13-week, randomized, placebo-controlled clinical trial in 125 children with ASD or SMS whose sleep problems had failed to be improved by behavioral intervention alone. Consequently, they found PedPRM effective, safe, and highly acceptable for the treatment of insomnia in studied children. Subsequently, Maras et al. [38] conducted a 39-week, open-label clinical study of PedPRM in 95 children with ASD or SMS subsequent to their prior 13-week randomized double-blind controlled clinical trial [39]. Consequently, Maras et al. reported that PedPRM was effective and safe for insomnia of studied children and improved caregivers’ quality of life as well. Interestingly, the proportions of completers were high in both the present study of immediate-release melatonin (93.9%) and these previous 2 studies of PedPRM (84.2% [38] and 85.0% [39]); this outcome is presumably attributable to the easily swallowed formulations of melatonin—granules and mini-tablets, respectively.

The present study has some limitations. First, the study was designed as an open-label study. Therefore, we could not definitely determine whether the beneficial effects found were entirely generated by melatonin treatment or were somewhat influenced by the spontaneous remission of sleep problems. This point is a challenge that needs to be addressed in a longer-term, randomized placebo-controlled clinical trial in the future. Second, sample size was relatively limited. Another study enrolling a greater number of children will serve for the corroboration of the findings from the present study. Third, our study did not provide any sufficient clinical evidence on the efficacy of melatonin treatment for children with NDDs who may have psychiatric disorders including schizophrenia and bipolar disorder.

## Conclusions

Melatonin treatment, which lasted for 26 weeks, yielded the following clinical outcomes: considerable reductions in NDD-emergent difficulties (e.g., sleep problems, aberrant behaviors, impaired daily life, and poor academic performance) of studied children who underwent adequate sleep hygiene interventions; and eventual improvements in the well-being and quality of life of both children and their caregivers through reductions in children’s manifestations (e.g., bad temper upon wakening) and caregivers’ burdens. In addition, melatonin treatment was well tolerated by studied children. Taken together, long-term melatonin treatment in combination with adequate sleep hygiene interventions may afford clinical benefits to children with NDDs and potentially elevates their well-being and causes a radical shift in current therapeutic approaches for them.

## Data Availability

The raw data generated and analyzed in this study are not publically available due to the appropriate protection of the personal protection of children and adolescents but are available from the corresponding author on a reasonable request.

## Abbreviations

ABC-J: Aberrant behavior check list-Japanese version
ADHD: Attention-deficit/hyperactivity disorder
AEs: Adverse events
ASD: Autism spectrum disorder
NDDs: Neurodevelopmental disorders
Q: Quartile
SMS: Smith-Magenis syndrome
SOL: Sleep onset latency
TEAEs: Treatment-emergent adverse events
